# *Cinnamomum zeylanicum* Extract and its Bioactive Component Cinnamaldehyde Show Anti-Tumor Effects *via* Inhibition of Multiple Cellular Pathways

**DOI:** 10.3389/fphar.2022.918479

**Published:** 2022-06-02

**Authors:** Sadhna Aggarwal, Kanchan Bhadana, Baldeep Singh, Meenakshi Rawat, Taj Mohammad, Lamya Ahmed Al-Keridis, Nawaf Alshammari, Md. Imtaiyaz Hassan, Satya N. Das

**Affiliations:** ^1^ Department of Biotechnology, All India Institute of Medical Sciences, New Delhi, India; ^2^ Center for Interdisciplinary Research in Basic Sciences, Jamia Millia Islamia, New Delhi, India; ^3^ Department of Biology, College of Science, Princess Nourah Bint Abdulrahman University, Riyadh, Saudi Arabia; ^4^ Department of Biology, College of Science, University of Hail, Hail, Saudi Arabia

**Keywords:** Cinnamomum zeylanicum, cinnamaldehyde, oral cancer, molecular docking, MAP kinase p38 alpha, dihydrofolate reductase Abbreviations: Cinnamomum zeylanicum extract (CZE), cinnamaldehyde (CIN), oral squamous cell carcinoma (OSCC), and dihydro folate reductase (DHFR)

## Abstract

*Cinnamomum zeylanicum* is a tropical plant with traditional medicinal significance that possesses antimicrobial, antifungal, anti-parasitic, and anti-tumor properties. Here, we have elucidated the anti-tumor effects of *Cinnamomum zeylanicum* extract (CZE) and its bioactive compound cinnamaldehyde (CIN) on oral cancer and elucidated underlying molecular mechanisms. Anti-tumor activities of CZE and CIN were demonstrated by various *in vitro* experiments on oral cancer cells (SCC-4, SCC-9, SCC-25). The cell proliferation, growth, cell cycle arrest, apoptosis, and autophagy were analyzed by MTT, clonogenic assay, propidium iodide, annexin-V-PI, DAPI, and acridine orange staining, respectively. The binding affinity of CIN towards dihydrofolate reductase and p38-MAP kinase alpha was analyzed by molecular docking. Western blot assay was performed to assess the alteration in the expression of various proteins. CZE and CIN treatment significantly inhibited the growth and proliferation of oral cancer cells in a dose-dependent manner. These treatments further induced apoptosis, cell cycle arrest, and autophagy. CZE and CIN inhibited the invasion and cytoplasmic translocation of NF-κB in these cell lines. CIN showed a high affinity to MAP kinase P38 alpha and dihydrofolate reductase with binding affinities of −6.8 and −5.9 kcal/mol, respectively. The cancer cells showed a decreased expression of various PI3k-AKT-mTOR pathways related to VEGF, COX-2, Bcl-2, NF-κB, and proteins post-treatment.

## Introduction

Oral cancer, a subtype of head and neck cancer, is a malignant neoplasm that arises onto the lip, the floor of the mouth, cheek lining, gingiva, palate, or tongue. It is one of most common cancer in India because of tobacco chewing habits (Yu et al., 2019; Kundu et al., 2022). Severe alcoholism and unchecked tobacco use like cigarettes, smokeless tobacco, betel nut chewing, poor dental care, poor diet, and human papillomavirus (HPV) are the most common risk factors for oral cancer (Ferlay et al., 2021; Chowdhury and Markus, 2022). The international agency for cancer research has predicted that India’s incidence of cancer will increase from 1 million in 2012 to more than 1.7 million in 2035. This indicates that the death rate will also increase from 680,000 to 1–2 million in the same period (Bray et al., 2013). In India or otherwise, 90–95% of oral cancer are squamous cell carcinoma (Nandi et al., 2021). Historically, the death rate associated with this cancer is particularly high because the cancer is routinely discovered late in its advanced stage, resulting in low treatment outcomes and higher costs. No significant advancement in the treatment of oral cancer has been found in recent years. Although the present treatments improve the quality of life of oral cancer patients, the overall survival rate of 5 years has not improved in the past few decades. Thus, the recent research towards generating therapies against oral cancer involves comparatively lesser side effects and cost-effective approaches.

Natural products are extensively used to target cancer therapy with minimal side effects (Anwar et al., 2020a; Anwar et al., 2020b; Anjum et al., 2021; Anwar et al., 2021; Anwar et al., 2022a; Anwar et al., 2022b; Anwar et al., 2022c). *Cinnamomum zeylanicum* (CZ) is a tropical evergreen plant belonging to the genus Cinnamomum. It is the major cinnamon and cinnamon oil used as spices and aromatics from ancient times. Besides its use as a food additive, it has cured gynecological, digestive, and respiratory illnesses. In various reports, Cinnamomum zeylanicum has antimicrobial effects against a broad range of pathogenic bacterium, *including Mycobacterium tuberculosis*, *Helicobacter pylori*, and *Streptococcus pneumoniae*. Similar studies have also elucidated its antifungal and anti-parasitic (Qaid et al., 2021; Moreno et al., 2022), and antidiabetic properties (Stevens and Allred, 2022). In addition, anti-tumor effects of *Cinnamomum zeylanicum* bark against various carcinomas are well established (Alsayadi et al., 2022).

*Cinnamomum cassia, Cinnamomum bumanni* and *Cinnamomum loureiroi* are the other varieties of cinnamon tree. But *Cinnamomum zeylanicum* is called “true cinnamon” because it has the highest content of trans CIN (49.9–62.8% of total bark oil) and the lowest coumarin content is carcinogenic and hepatotoxic. Besides trans-CIN, eugenol and linalool are also the major components of *Cinnamomum zeylanicum*. Trans-cinnamaldehyde, eugenol and linalool all together represent 82.5% of the total composition of *Cinnamomum zeylanicum* (Albuquerque et al., 2021). CIN is the bioactive component of Cinnamon extract. Various reports have shown its activity against different pathogens (Neto et al., 2022). Further, it showed anti-cancer effects against various cancers (Chang et al., 2021; Kumari and Singh, 2021).

MAP kinase P38 alpha (MAPKP-38α) is a member of mitogen-activated protein kinases activated by various stimuli like heat shock, oxidative stress, inflammatory cytokines, damaged DNA, etc., (Zeyen et al., 2022). It regulates cellular functions like division, stress response, differentiation, survival, and immune (García-Hernández et al., 2021). The activation of MAPKP-38α by reactive oxygen species (ROS) in cancer cells ultimately leads to the apoptosis of cancer cells (Dolado et al., 2007). Despite its role in cancer suppression, numerous studies have demonstrated its involvement in cancer progression (Kennedy et al., 2007). A higher expression of MAPKP-38α in oral squamous cell carcinoma (OSCC) was observed with a mild decrease in tumor growth while inhibiting MAPKP-38α with a pharmacological agent, SB2023580 (Gkouveris et al., 2020). MAPKP-38 could be considered a promising drug target for treating OSCC (Chung et al., 2022).

On the other hand, dihydrofolate reductase (DHFR) is an enzyme that catalyzes the reduction of dihydrofolate to tetrahydrofolate, which ultimately acts as a cofactor for the *de novo* biosynthesis of purines, thymidylate and certain amino acids (Shamshad et al., 2022). Because of its involvement in synthesizing various components required for cell proliferation, it has been used as a drug target for cancer treatment. Methotrexate is the key example of an anti-cancer drug that inhibits DHFR (Srinivasan et al., 2019). PI3K/Akt/mTOR is the key signaling pathway regulating cell proliferation, apoptosis, and differentiation. Inhibition of cancer progression by inhibiting PI3K/Akt/mTOR signaling pathway in various cancers such as prostate (Braglia et al., 2020), bladder (Li et al., 2022), breast (Song et al., 2022) and oral cancers (Marques et al., 2022).

Molecular docking is a powerful computational technique in structural biology and computer-aided drug design used in the process of drug discovery to identify small drug-like compounds by predicting their binding mode and affinities to a biological target (Naqvi and Hassan, 2016; Naqvi et al., 2018; Naqvi et al., 2020). Today, molecular docking is one of the most important components of modern drug discovery research, which includes several computational methods that allow researchers to predict the binding prototype of a molecule to a receptor. In general, this technique considers a theoretical approach, which is used to save the cost, time, and resources needed to perform compound screening experimentally in wet-lab conditions to facilitate lead discovery (Queen et al., 2018; Mohammad et al., 2020; Jairajpuri et al., 2021; Roy et al., 2021).

In the present study, we have explored the anti-tumor potential of *C. zeylanicum* extract and its bioactive component on Oral cancer cell line. In addition, we have performed a series of cell-based assays to explore the apoptotic potential of CZE and CIN towards Oral cancer cell line (Yu et al., 2019). We performed molecular docking of CIN with MAPKP-38α and DHFR to predict their bound conformations and binding affinities. Finally, we elucidated the underlying molecular mechanisms that might be responsible for these effects by finding out the significant binding protein and then studying the alteration in expression of different proteins that are involved in apoptosis (NF-kB and P53), cell cycle (Cyclin D1) and angiogenesis (Vascular endothelial growth factor, VEGF) leading to tumor spread and establishment.

## Materials and Methods

### Oral Cancer Cell Line Culture, Maintenance and Treatment

This study was performed SCC-4, SCC-9, and SCC-25 oral cancer cell lines procured from Prof. Martin R Berger, DKFZ, Germany. The details of the cell lines have been described elsewhere (Aggarwal et al., 2015). These cells were maintained in Dulbecco’s Modified Eagle’s Medium (DMEM) supplemented with 10% fetal bovine serum (FBS), 1% sodium pyruvate, 100 U/ml penicillin-G and 100 μg/ml streptomycin (all from HiMedia corporation Mumbai, India) at 37°C in humidified 5% CO_2_ under sterile conditions. Pilot experiments were performed to determine the IC50 doses of CZE and CIN at 24, 48 and 72 h of treatment in oral cancer cell lines. However, subsequent experiments were performed at varying concentrations of drugs at 48 h only. Each experiment was repeated thrice, and the untreated tumor cells served as a control in all the experiments.

### Preparation of *C. zeylanicum* Extract

A certificate of authenticity for the bark of *C. zeylanicum* was obtained from CSIR-NISCAIR (New Delhi, India), and the hydro-methanolic extract of powdered bark was prepared as described previously (Budiastuti et al., 2021). Briefly, coarse powder of the bark was incubated overnight with 50% ethanol in an orbital incubator shaker. The filtrate extracted was lyophilized. The aliquots of powdered extract were preserved at −20°C. The percentage yield was calculated based on the weight of initial and final dried plant material used for extract preparation. The powdered extract was resuspended in 1% DMSO to obtain the desired concentration for further experiments. Commercially available Cinnamaldehyde (Sigma Aldrich, United States) was used simultaneously for all the experiments.

### Isolation of Peripheral Blood Mononuclear Cells

Heparinized peripheral blood was collected from normal individuals by venipuncture and diluted 1:2 with fresh sterile phosphate-buffered saline (PBS). PBMCs were isolated by density gradient centrifugation using Histopaque-1077 (Sigma-Aldrich, St. Louis, MO, United States). A trypan blue dye exclusion test determined the viability of the cells as described earlier (Aggarwal and Das, 2016).

### Assessment of Tumor Cell Growth by MTT Assay

Effects of drugs on tumor cell growth inhibition were determined by MTT [3-(4,5-dimethylthiazol-2-yl)-2,5-Diphenyltetrazolium bromide] dye reduction assay as described earlier (Aggarwal et al., 2014). Briefly, 8×10^3^ cells per 100 µL DMEM per well were seeded in triplicates in 96-well plates for 16 h at 37°C in a CO_2_ incubator. The synchronized cells were then treated with different concentrations of the drugs for 24, 48 and 72 h. Human PBMCs were used as normal controls simultaneously. After treatment, the wells were observed under an inverted light microscope for capturing morphological changes. For MTT assay, 10 µL of MTT solution (Sigma Aldrich, United States; 10 mg/ml) was added to each well and the plate was further incubated for 4 h at 37°C in CO_2_ incubator. The formazan crystals formed were dissolved by DMSO. The number of formazan crystals formed, i.e., color intensity, was measured by spectrophotometer at A_570nm_ and % proliferation was calculated using the following formula:

$$\%\;\mathrm{Proliferation=}\left(\mathrm{1-O}D_{\mathrm{test}}\mathrm{/O}D_{\mathrm{control}}\right)\mathrm{\times 100}$$

The experiment was repeated thrice with the same passage number of the cell line. The data represent mean ± SD of three independent experiments.

### Clonogenic Assay

The clonogenic assay was also performed to assess the effects of the drug on the proliferation of oral cancer cells. As described earlier, the assay was performed (Aggarwal et al., 2015). Briefly, the tumor cells (500 cells/well) were cultured in a six well tissue culture plate and incubated at 37°C, 5% CO_2_ in humidified air for 16 h. Then, three increasing concentrations of drugs (i.e.,<IC 50, IC 50, > IC 50) were added to the respective wells and the plates were re-incubated for 5–10 days. The media was changed every alternate day and the plates were observed for colony (clusters of 20 or more cells) formation under an inverted microscope. The acetone-fixed colonies were then stained with 0.5% crystal violet solution and scored. The data has been represented as %survival, i.e., (Number of colonies after treatment/Number of tumor cells seeded) X100.

### Cell Cycle Analysis by Flow Cytometry

The effect of drugs on cell cycle progression was analyzed by propidium iodide (Sigma Aldrich, United States) labeling followed by flow cytometry as described earlier (Aggarwal and Das, 2016). Briefly, tumor cells with CZE and CIN for 48 h in six well plates. Trypsinised cells were then washed twice with PBS and fixed in chilled 70% ethanol on ice for 4 h. After a PBS wash, cells were treated with 1 mg/ml RNase A (Sigma Aldrich, United States) for 30 min at 37°C. Finally, the cells were incubated with 50 μg/ml propidium iodide (Sigma Aldrich, United States) for 10 min and acquired on a flow cytometer (LSRII, BD Biosciences, CA, United States). The distribution of cells in the cell cycle’s G0/G1, S and G2/M phases was determined using the ModFit LT software.

### Apoptosis Analysis by AnnexinV- Binding Assay

The ability of CZE and CIN to induce apoptosis in oral cancer cell lines was assessed by annexin-V- binding assay as described earlier (Aggarwal and Das, 2016). Briefly, the cells were treated as in previous experiments. PBS washed cells were resuspended in 100 μL annexin-V binding buffer. Five microliters of FITC-conjugated annexin-V (BD Biosciences, CA, United States) and 50 μg/ml of PI were added to the cell suspension and further incubated for 15 min at room temperature. The cells were acquired on a flow cytometer (LSRII, BD Biosciences, CA, United States) and the data was analyzed using BD FACSDiva™ software (BD Biosciences, CA, United States).

### Nuclear Staining by 4-6-Diamidino-2-Phenylindole

The effects of drug treatment on apoptosis were also studied by staining the nucleus with DAPI (4-6-Diamidino-2-Phenylindole). The drug-treated cells were fixed and permeabilized using 4% paraformaldehyde and ice-cold methanol: acetone (1:1). The cells were mounted with Fluoroshield (Sigma- Aldrich St. Louis, MO, United States), containing the DAPI. The slides were observed under Nikon Eclipse E600 Microscope and images were obtained using NIS- elements microscope imaging software (Nikon, Tokyo, Japan).

### Acridine Orange Staining for Detection of Acidic Vesicular Organelles

The tumor cells (SCC-4) were plated on a coverslip in 12 well plates and allowed to adhere by incubating overnight in CO_2_ incubator at 37°C. Next day, the cells were treated with the IC_50_ concentration of CIN. After 48 h, cells were washed with 1X PBS and stained with 1 μg/ml Acridine orange solution for 15 min, followed by fixation with 4% paraformaldehyde for 20–30 min. Excessive PFA was washed and coverslips were mounted onto the slides. Slides were observed at 488 and 568 nm excitation filter under a fluorescence microscope at ×40 magnification (Zeiss, Germany). The images obtained at 488 and 568 nm excitation wavelengths were merged using Zeiss software.

### NF-κB Nuclear Translocation Assay

The tumor cells (SCC-4) were treated with IC_50_ dose of CIN for 48 h at 37°C in a CO_2_ incubator. The untreated and treated cells were collected after trypsinization, fixed in 4% paraformaldehyde and permeabilized by 1:1 cold methanol/acetone. The cells were then incubated with Rabbit anti-human pNF-κB/p65 antibody (Aggarwal and Das, 2016). After washing with 1X PBS, cells were labeled with anti-rabbit IgG (H + L)-Alexa Flour^®^ 488 (Invitrogen, Ma, United States)**.** The nucleus was stained with DAPI stain at 300 nm concentration. Slides were then observed under a fluorescence microscope at ×40 magnification (Zeiss, Germany).

### Cell Invasion Assay Using Matrigel

The cell invasion capability of tumor cells after treatment was observed by cell invasion assay using matrigel as described previously (Aggarwal et al., 2019). Firstly, the thawed (4°C overnight) matrigel was diluted to a working concentration (1 mg/ml) in serum free-cold cell culture media (DMEM). It was then layered onto the upper chamber, i.e., transwell, placed in a 24-well plate and incubated at 37°C for at least 4–5 h for polymerization. Meanwhile, the drug-treated cells were harvested by trypsinization and washed thrice with culture media. Cells (5 × 10^4^) were seeded over the solidified layer of matrigel. The lower chamber, i.e., the well in a 24-well plate, was filled with 600 µL of culture media and incubated at 37°C for 20–24 h. After the incubation, the matrigel layer was carefully scraped off (un-invaded cells) with a cotton swab. The invaded cells at the bottom of the transwell were fixed and stained as in the clonogenic assay. The invaded cells were then counted under a light microscope, and the data was represented as % invasion, i.e., (number of cells invaded/number of cells seeded) X100.

### Immuno-Blotting and Enhanced Chemiluminescence Assay

Alteration in the expression of certain significant proteins by tumor cells after drug treatment was observed by western blot assay. The washed cell pellet of treated cells was lysed in chilled RIPA buffer (Sigma Aldrich, United States) and a 1X protease inhibitor cocktail (Sigma Aldrich, United States) on ice for 30 min with intermittent mixing by three freeze-thaw cycles. Lysates were clarified by centrifugation and aliquots were stored at −80°C. As described elsewhere, immunoblotting was performed (Aggarwal et al., 2015). An equal number of proteins were resolved by SDS-PAGE and electrotransferred onto nitrocellulose membrane (Millipore Pvt. Ltd., India). 5% BSA blocked membrane was washed with wash buffer (Wash buffer: 20 mm TrisCl, 500 mm NaCl, 0.05% v/v tween 20). Blots were then incubated with respective primary antibodies [anti-human pNF-κB/p65, COX-2, cyclin D1, VEGF, P110a, AKT, mTOR, P-mTOR, and β-actin antibodies (Cell Signalling Technology, Boston, MA, United States); Cyclin D1 (Abcam, MA, United States), Beclin-1 (BD Pharmingen, United States)] for overnight at 4°C. After washing thrice, the membranes were incubated with host-specific HRP conjugated secondary antibody (Cell Signalling Technology, Boston, MA, United States) at room temperature 45 min. Immunoreactive bands were detected by enhanced chemiluminescence procedure using ECL kit (Thermo Fischer Scientific, MA, United States) and image was acquired by a gel imaging system (Protein Simple, Santa Clara, CA, United States). The band intensity was measured by using the “ImageJ” software (NIH, United States).

### Molecular Docking of Cinnamaldehyde With MAP Kinase p38 Alpha and DHFR

Three-dimensional coordinates of MAPKP38α and DHFR were taken from Protein Data Bank (PDB ID: 3ZS5 and 3GHW, respectively) (Azevedo et al., 2012; Zaware et al., 2017). The molecular structure of CIN was downloaded from PubChem and pre-processed using the Open Babel module to facilitate structure-based molecular docking in Instadock (Mohammad et al., 2021). For visualization purposes, PyMOL and Discovery Studio visualizers were employed. Apart from the visualization, measurements like bond length, the distance between two coordinates, and the distance between nucleotide and ligand were calculated using these tools. Online resources such as PubChem, PDB, SwissADME, etc., were used in retrieval, evaluation and analysis.

### Statistical Analysis

All experiments were performed in triplicates, and the results were expressed as mean ± standard deviations. The significance of the difference between the two variables was analyzed by paired t-test using GraphPad PRISM version 6.0 (La Jolla, CA, United States). The *p*-value of <0.05 was considered to be statistically significant.

## Results

### *CZE* and CIN Induce Cytotoxicity in OSCC Cells

**FIGURE 1:**
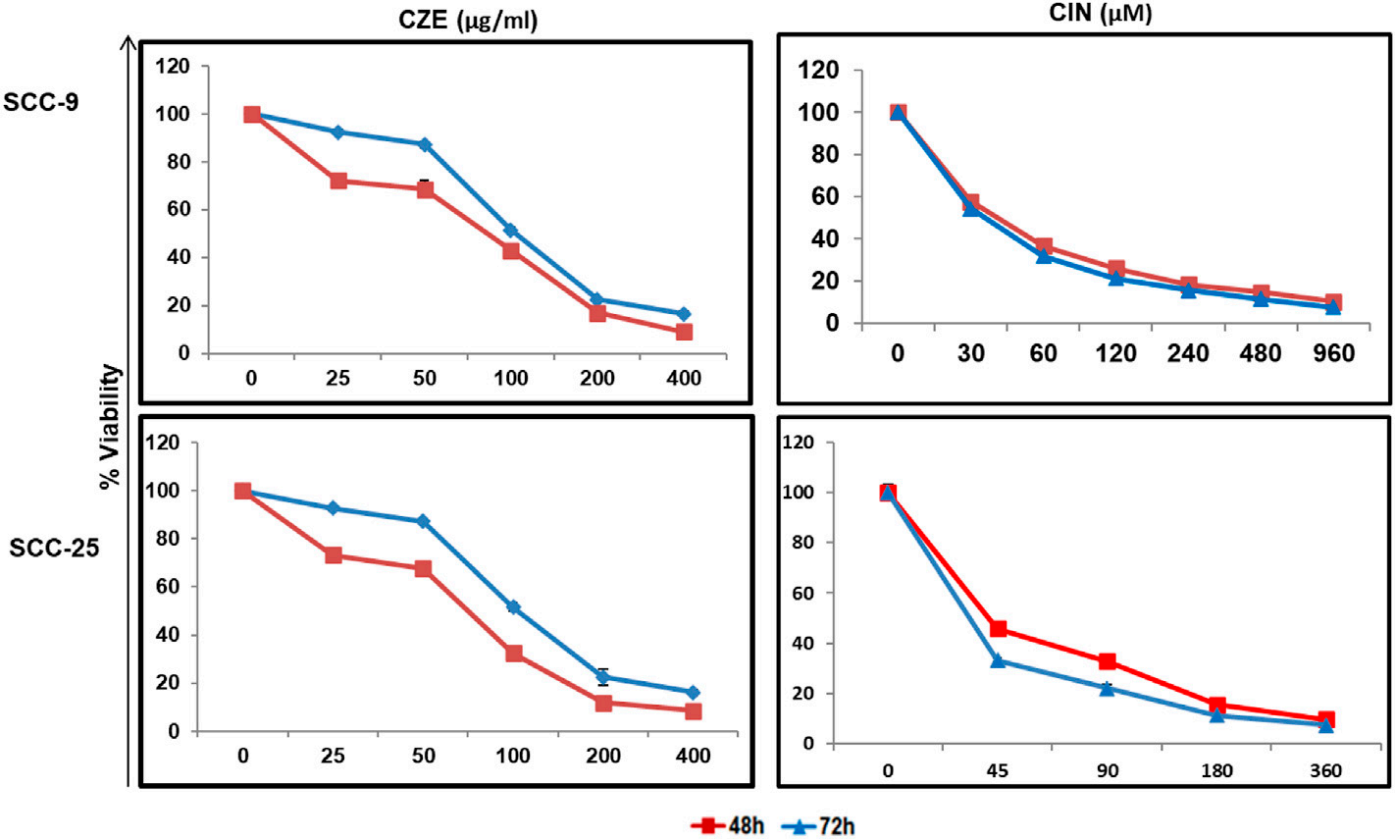
Dose response curves showing cytotoxicity in oral cancer cells by CZE and CIN treatment. MTT assay showing dose dependent decrease in the viability of SCC-9 and SCC-25 cells after CZE (0–400 μg/ml) and CIN (0–960 μm) at 48 and 72 h of treatment. Each value in line graph clearly shows the significant decrease in the viability with increasing extract concentration as compared with untreated cells. The data has been represented as the mean ± SD of three independent experiments.

**FIGURE 2:**
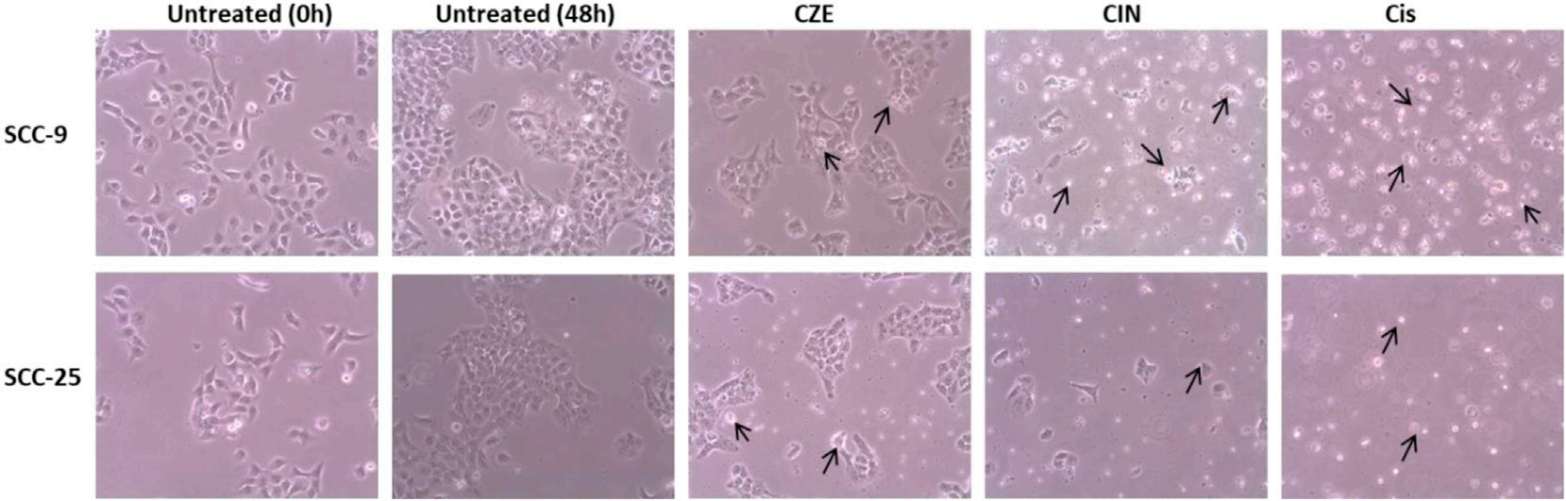
Photomicrographs showing morphological changes in OSCC cells after CZE and CIN treatment at 48 h. As shown by arrows, the cells were smaller, detached from culture plates, lost their original shape and showed membrane blebbing. *Cinnamomum zeylanicum* extract (CZE), cinnamaldehyde (CIN) and Cisplatin (Cis). CZE and CIN inhibited the colony-forming potential of OSCC cells.

The cytotoxic effect of the CZE and CIN on OSCC cells was studied by performing an MTT assay. A dose-dependent decrease in tumor cell viability was observed after treatment with CZE (0–400 μg/ml) and CIN (0–960 µm) treated SCC-9 cells **(** Figure 1 **)**. A similar pattern was observed in SCC-25 cells post- CZE (0–400 μg/ml) and CIN (0–960 µm) treatment. The IC_50_ doses of CZE SCC-9-48 h (100 μg/ml) and 72 h (75 μg/ml); SCC-25-48 h (30 μg/ml) and 72 h (85 μg/ml) and CIN [SCC-9-24 h (120 μm), 48 h (40 μm) and 72 h (35 μm); SCC-25-24 h (250 μm), 48 h (45 μm) and 72 h (37 μm)] were derived from the dose-response curve. All the further experiments were performed at the gradient of IC50 doses at 48 h. As shown in Figure 2, microscopic observation revealed significant morphological changes in OSCC cells after the drug treatment, such as detachment and cell shrinkage. Also, the healthy PBMCs showed minimal cytotoxicity (<5%) after CZE and CIN treatments (data not shown).

**FIGURE 3:**
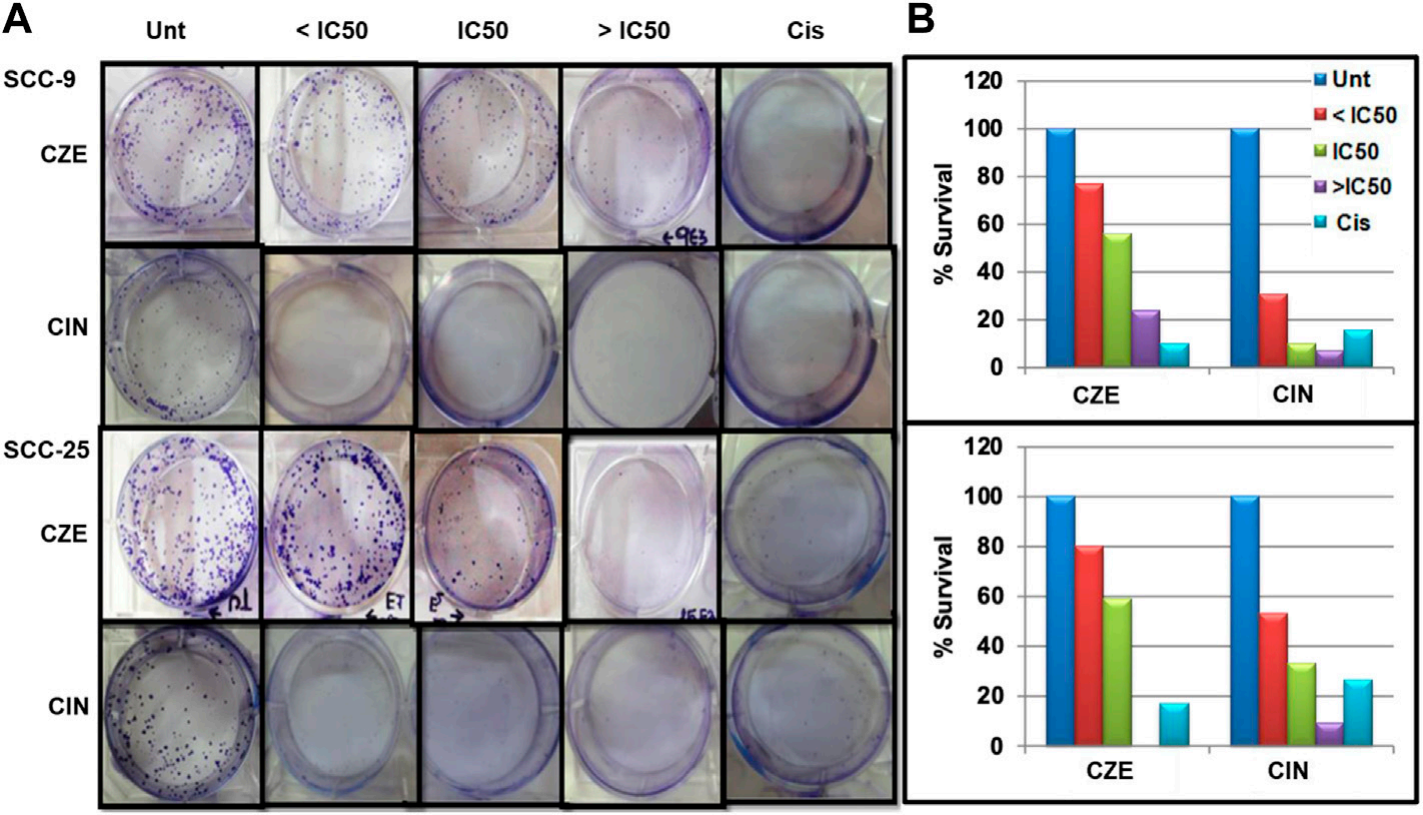
Clonogenic assay **(A)** Representative images of colony forming assay after treatment with CZE and CIN on SCC-9 and SCC-25 cells at 48 h **(B)** Bar graph showing % CFU of cancer cells after treatment with CZE and CIN. *Cinnamomum zeylanicum* extract (CZE), cinnamaldehyde (CIN) and Cisplatin (Cis).

To investigate the potential cell proliferative inhibition activity of CZE and CIN in oral cancer. The effect of CZE and CIN was examined on clonogenic survival by *In-vitro* clonogenic assay. A significant dose-dependent reduction in % CFU was observed in SCC-9 and SCC-25 cell lines after treatment with ± IC_50_ of CZE and CIN at 48 h (Figure 3). In SCC-9 maximal proliferation, inhibition was observed at 48 h with 200 μg/ml CZE and 80 µm CIN, which inhibited the colony growth from 100 to 24% and from 100 to 7.27%, respectively. Similarly, in the case of SCC-25 maximal proliferation inhibition was observed at 48 h with 240 μg/ml *CZE* and 90 µm CIN, which inhibited colony growth from 100 to 0% and from 100 to 7.27%, respectively.

### *CZE* and CIN Induce S and G2/M Arrest in Oral Cancer Cells

**FIGURE 4:**
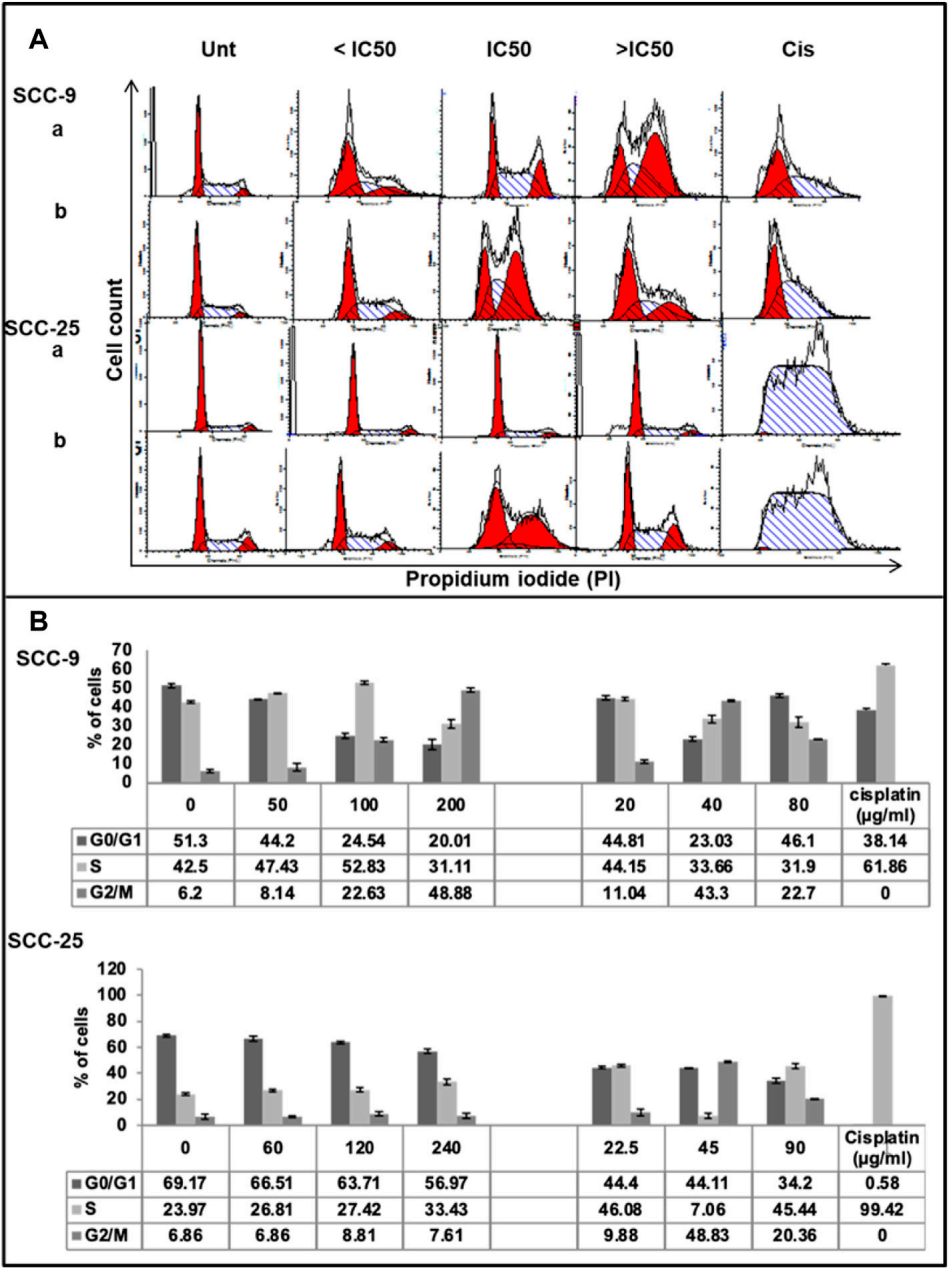
Effect of CZE and CIN on cell cycle of oral cancer cells. Flow cytometric analysis of DNA content in different phases of the cell cycle after 48 h exposure to the indicated concentrations of **(a)**. CZE and **(b)**. CIN on both SCC-9 and SCC-25 cells **(A)** Representative histogram cell cycle analysis of untreated, CZE (50, 100, and 200 μg/ml) and CIN treatments (20, 40, and 80 μm) and cisplatin (2 μg/ml) treated cancer cells **(B)** Bar graphs showing the percentage (each value represents as mean ± SD) of cells in each phase indicating a significant arrest in specific phase of cell cycle.

Cell cycle analysis assay was performed to observe the effect of CZE and CIN treatment on the cell cycle progression of OSCC cells (Figure 4). The results were obtained by analyzing the distribution of the percentage of PI + cells in different cell cycle phases, i.e., propidium iodide staining assay followed by flow cytometric analysis. A dose-dependent increase in the percentage of PI^+^ cells was observed in the S and G2/M phase of the cell cycle after CZE and CIN treatment in SCC-9 and SCC-25 cells.

CZE treatment induced the accumulation of cells in the S-phase (from 47.62 to 52.83%) and G2/M phase (from 8.26 to 48.83%), while CIN treatment induced G2/M phase arrest (from 6.2 to 22.7%) in SCC-9 cells. Similarly, CZE induced S-phase arrest (from 23.97 to 33.43%) and CIN induced S- phase (from 38.06 to 45.44%) and G2/M phase (from 13.84 to 20.36%) arrest in SCC-25 cells. Positive control, cisplatin, induced significant arrest in S-phase in both the oral cancer cell lines.

### *CZE* and CIN Induce Early and Late Phase Apoptosis in Oral Cancer Cells

**FIGURE 5:**
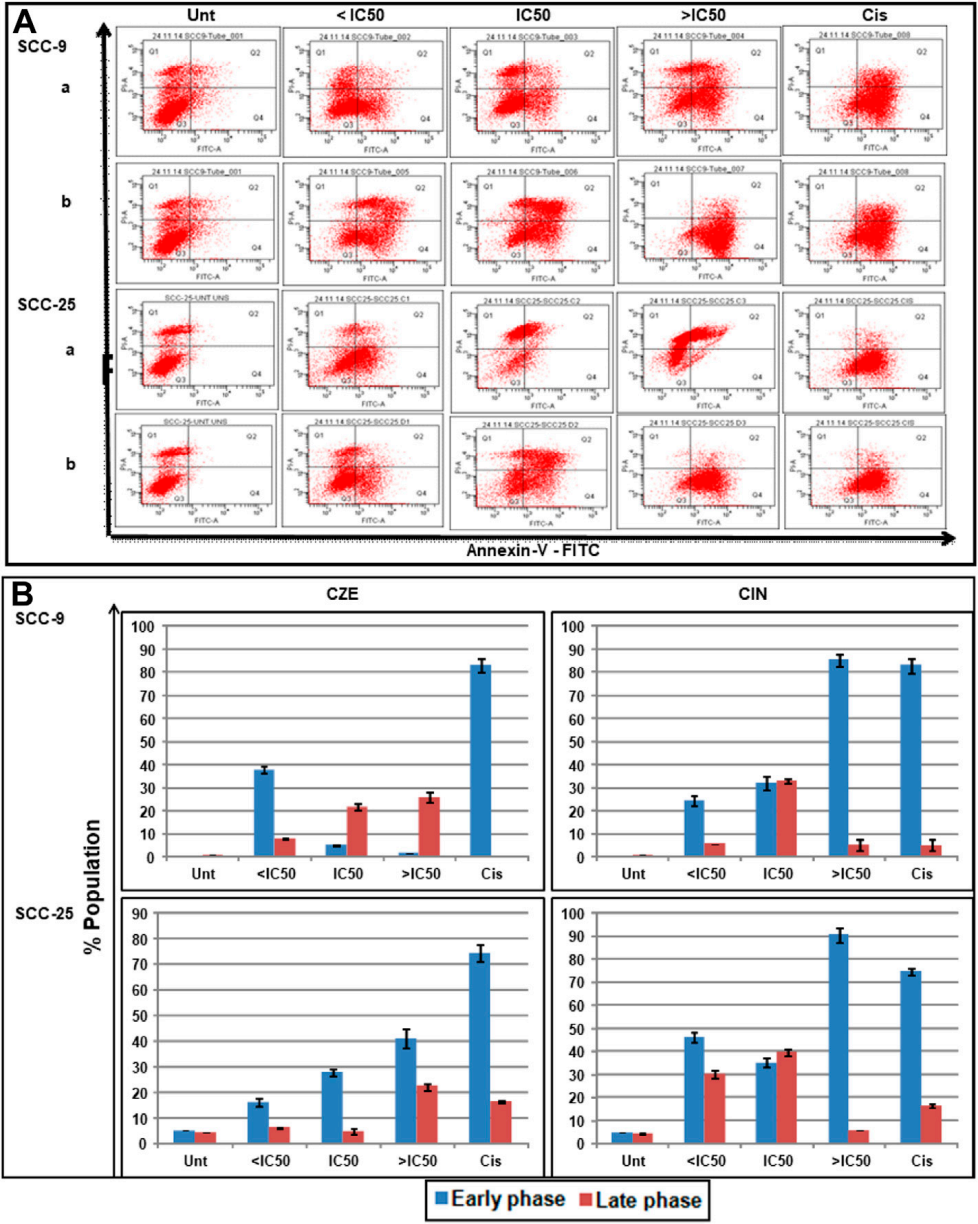
Apoptotic effects of CZE and CIN on oral cancer cells as observed Annexin-V-FITC double staining. The untreated, CZE (50, 100, and 200 μg/ml), CIN (20, 40, and 80 μm) and cisplatin (2 μg/ml) treated cancer cells (SCC-9 and SCC-25) cells were subjected to Annexin-V-FITC double staining at 48 h **(A)** Representative quadrant plots from flow cytometry analysis showing dose-dependent increase in apoptotic cells. Quad I (Top-left): Necrotic (Annexin-V-FITC^-^/PI^+^); Quad II (Top-right): late apoptotic cells (Annexin-V-FITC^+^/PI^+^); Quad III (Bottom-left): live cells (Annexin-V-FITC^-^/PI^−^); Quad IV (Bottom-right): early apoptotic cells (Annexin-V-FITC^+^/PI^−^) **(B)** Bar graphs showing the percentage distribution of cancer cells (mean ± SD) in various phases of apoptosis after treatment with different concentrations of Cinnamomum zeylanicum extract (CZE), cinnamaldehyde (CIN) and Cisplatin (Cis).

DAP-I and Annexin-V/PI staining assays studied the effects of *CZE* and CIN on apoptosis of oral cancer cells was studied by DAP-I and Annexin-V/PI staining assays. The annexin-V-FITC/PI double staining concluded the dose-dependent induction of apoptosis in oral cancer cells after CZE and CIN treatment compared to the untreated cells**.** In SCC-9 cells, the CZE treatment induced early phase (from 5.0 to 41.1%) apoptosis; while CIN showed early (from 5.0 to 90.5%) and late (6.2–39.7%) phase apoptosis. Similar trends were observed in SCC-25 cells, as shown in Figure 5.

**FIGURE 6:**
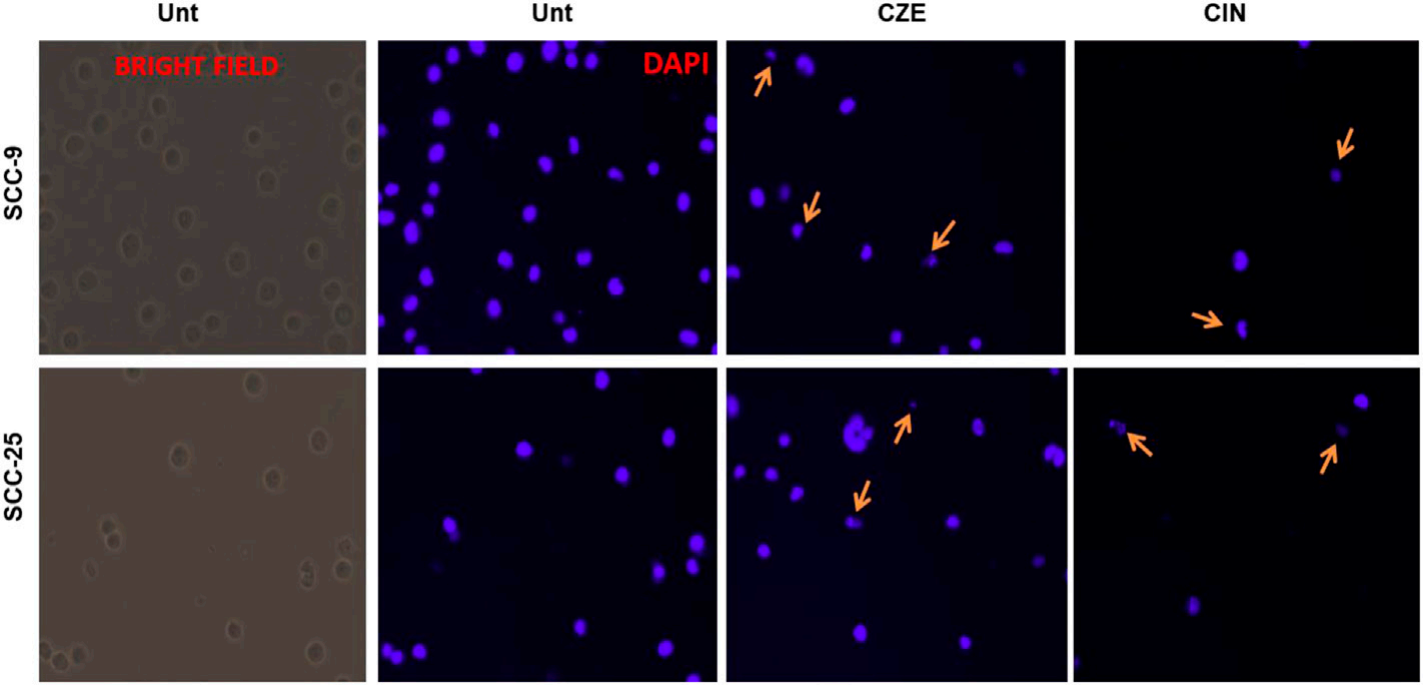
Photomicrographs showing DAP-I Staining The untreated SCC-9 and SCC-25 cells showed normal nuclear morphology, whereas CZE and CIN treated cells underwent nuclear fragmentation and cell shrinkage (as shown by arrows) (40X). CIN treatment induces autophagy in oral cancer cells.

Also, the OSCC cells showed the characteristic features of the apoptotic nucleus after DAP-I staining on treatment with IC_50_ doses of *CZE* and CIN, i.e., the nucleus of treated cells showed fragmentation and shrinkage compared to untreated cells **(** Figure 6 **)**.

### Cinnamaldehyde Treatment Induces Autophagy in Oral Cancer Cells

**FIGURE 7:**
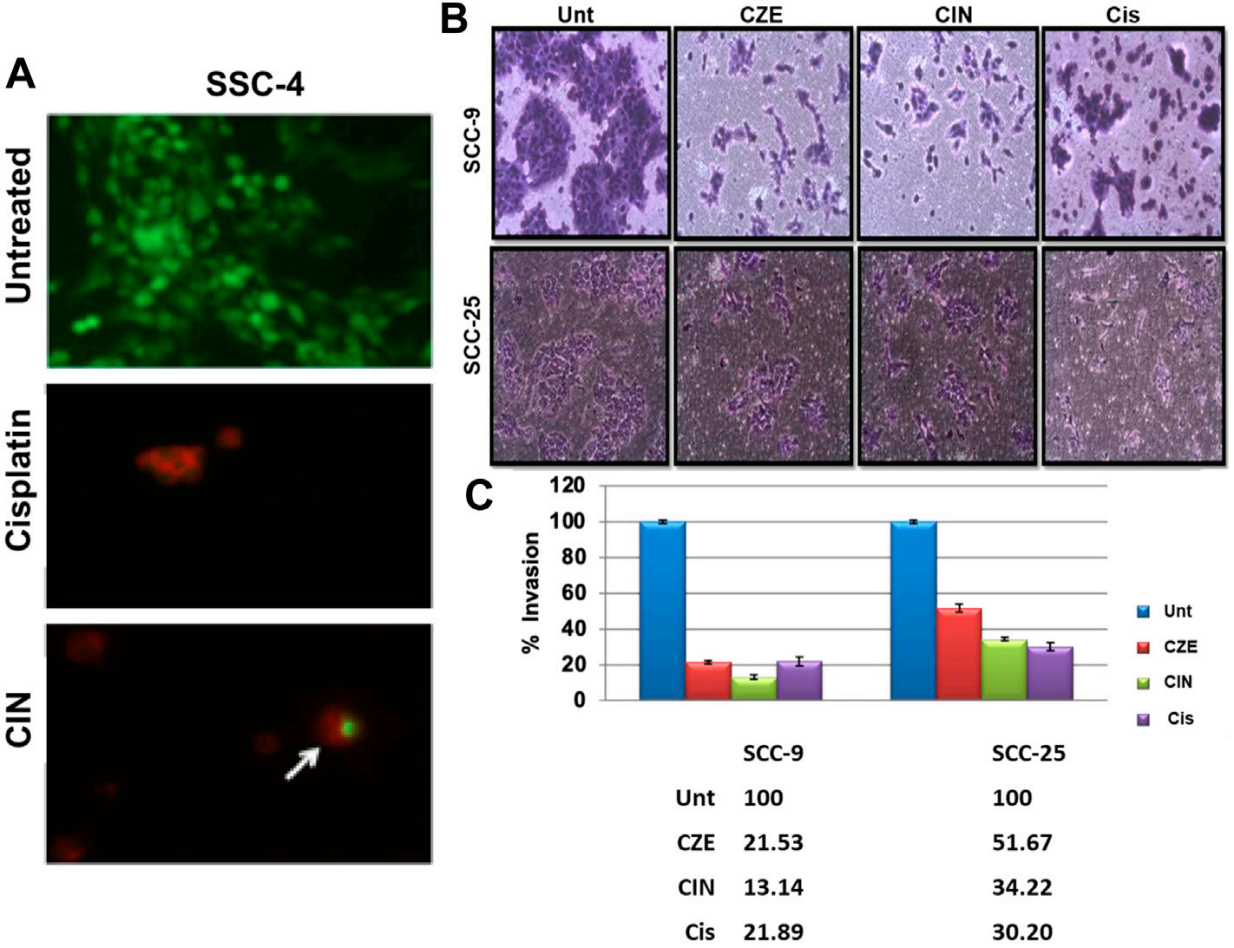
**(A)** Acridine orange staining showing acidic vesicular organelles (AVOs) in Cinnamaldehyde treated SCC4 cells. Arrows indicate the presence of bright red fluorescing autophagic vacuoles in the cell cytoplasm. No such vacuoles can be seen in the untreated SCC-4 cells **(B)** Matrigel assay showing decreased invasiveness of SCC-9 and SCC-25 cells post CZE and CIN treatment. Representative pictures of crystal violet stained untreated, CZE, CIN and cisplatin treated oral cancer cells (SCC-9, SCC-25) at 48 h. The experiment was performed in duplicates and three fields were counted for each chamber **(C)** Bar graphs showing a dose-dependent decrease in percentage invasion (Mean ± SD) of extract treated and CIN treated cancer cells. CZE and CIN treatment inhibited the migration of OSCC cells.

Autophagy was analyzed using acridine orange staining of acidic vesicular organelles (AVOs), including autophagic vacuoles. The cytoplasm and nucleus fluoresced bright green and dim red in untreated cells. The SCC-4 cells post cinnamaldehyde showed the presence of AVOs **(** Figure 7A), as shown by the concentrated bright red fluorescence in acidic compartments.

### CZE and CIN Treatment Inhibited the Migration of OSCC Cells

Invasion of endothelial cells to the basement membranes is an important step of angiogenesis, a hallmark of cancer. Hence, the effects of drug treatment on the migration ability of oral cancer cells were studied. CZE and CIN treatment decreased the migration or invasiveness of SCC-9 and SCC-25 cells into the matrigel (Figures 7B,C).

### Cinnamaldehyde Inhibits Nuclear Translocation of NF-κB in Oral Cancer Cells

**FIGURE 8:**
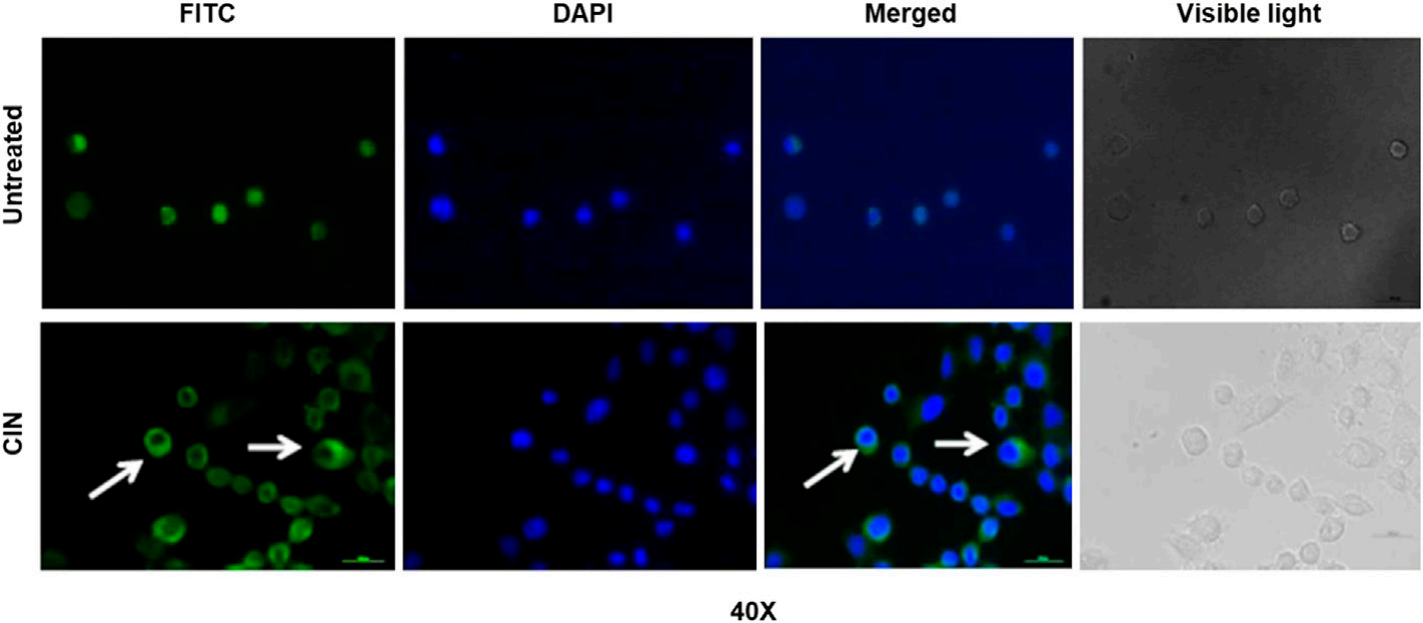
The photomicrographs showing expression of pNF-κB/p65 protein in SCC-4 cells. The picture shows the expression of pNF-κB/p65 protein in untreated and CIN treated SCC-4 cells. In the untreated cells the expression of pNF-κB/p65 protein (green) can be seen in cytoplasm and nucleus (blue). However, in the treated cells the accumulation of pNF-κB/p65 protein (green, shown by arrows) can be seen in the cytoplasm (blue: DAPI stain, green: Alexaflour^®^488).

The intracellular immunostaining of NF-κB/p65 in CIN SCC-4 was shown to inhibit the nuclear translocation of NF-κB/p65 (Figure 8). In untreated tumor cells, NF-κB protein (green color) was majorly localized in the nucleus (blue), whereas in CIN (IC_50_) treated SCC-4 cells, NF-κB was majorly localized in the cytoplasm.

### CZE and CIN Modulated the Expression Levels of Cancer-Related Proteins in Oral Cancer Cells

Western blotting was performed to investigate the effect of CZE and CIN on the expression of key regulatory proteins involved in cancer pathogenesis (Supplymentary Figure S1). Densitometric analysis revealed the decreased expression levels of NF-κB/p65, COX-2, p110a, CyclinD1, VEGF, AKT, T-mTOR, Ser2448p mTOR, and Bcl-2 after treatment with CZE and CIN, in both SCC-9 and SCC-25 cells. However, the beclin-1 expression was upregulated in response to CZE and CIN treatment in these cells. β-actin was taken as an internal loading control.

### Molecular Docking of cinnamaldehyde With MAPKP-38α and DHFR

The docking study observed that the CIN shows appreciable binding affinities towards MAPKP-38α and DHFR. CIN shows < −6.8 kcal/mol and < −5.9 kcal/mol binding affinities towards MAPKP38α and DHFR, respectively (Table 1). CIN shows a higher binding affinity with MAPKP38α than DHFR.

**TABLE 1:** CIN’s binding affinity with MAP kinase p38 alpha and DHFR.

S. No.	Target	Affinity (Kcal/mol)
1.	MAP Kinase p38 alpha	−6.8
2.	Dihydro Folate Reductase	−5.9

**FIGURE 9:**
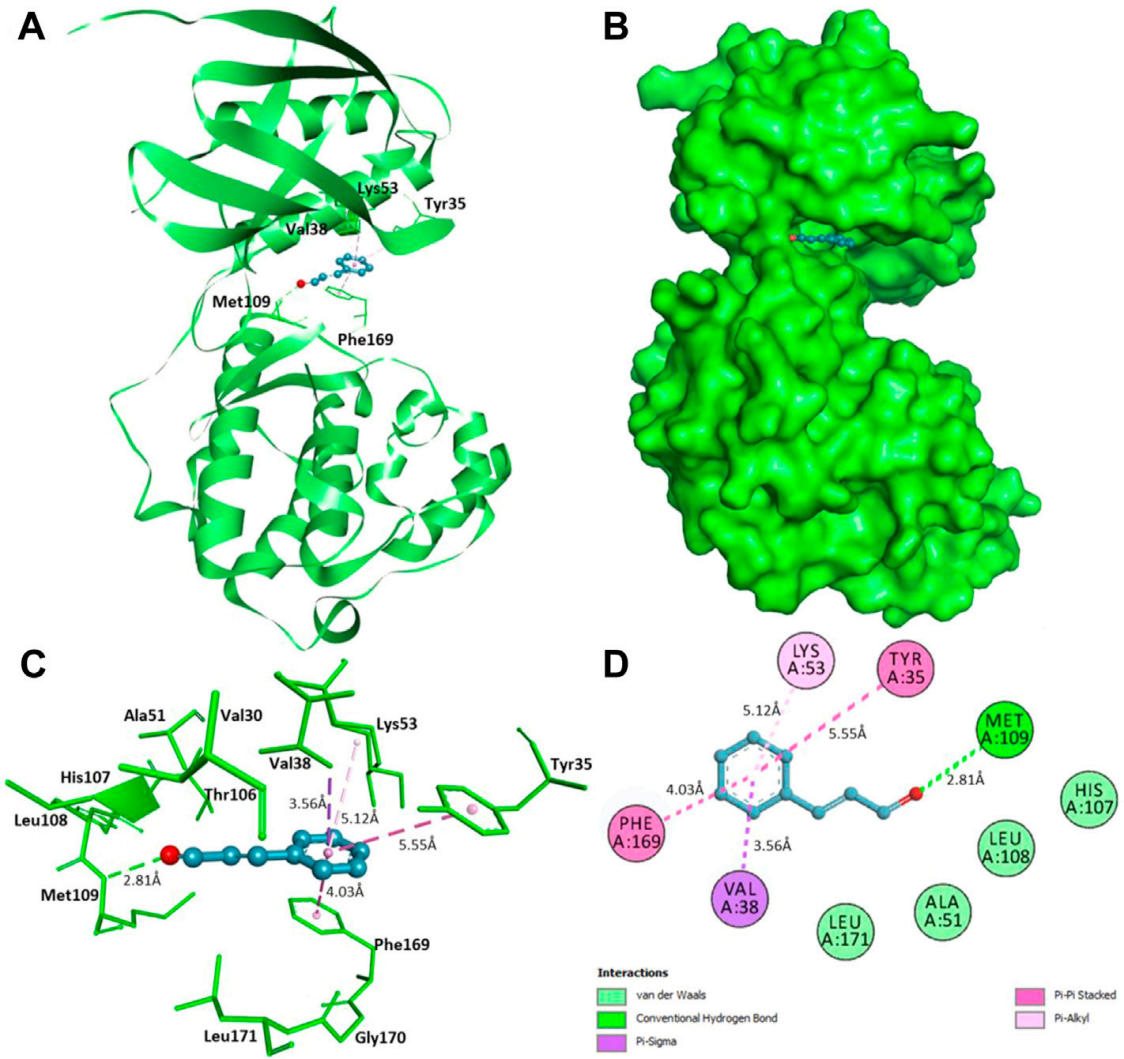
Interaction of CIN to MAPKP-38α **(A)** Cartoon representation of MAPKP-38α in-complexed with CIN **(B)** Surface view of MAPKP-38α in-complexed with CIN **(C)** 3D representation of interacting residues of MAPKP-38α with CIN **(D)** 2D plot of interacting residues of MAPKP-38α with CIN.

**FIGURE 10:**
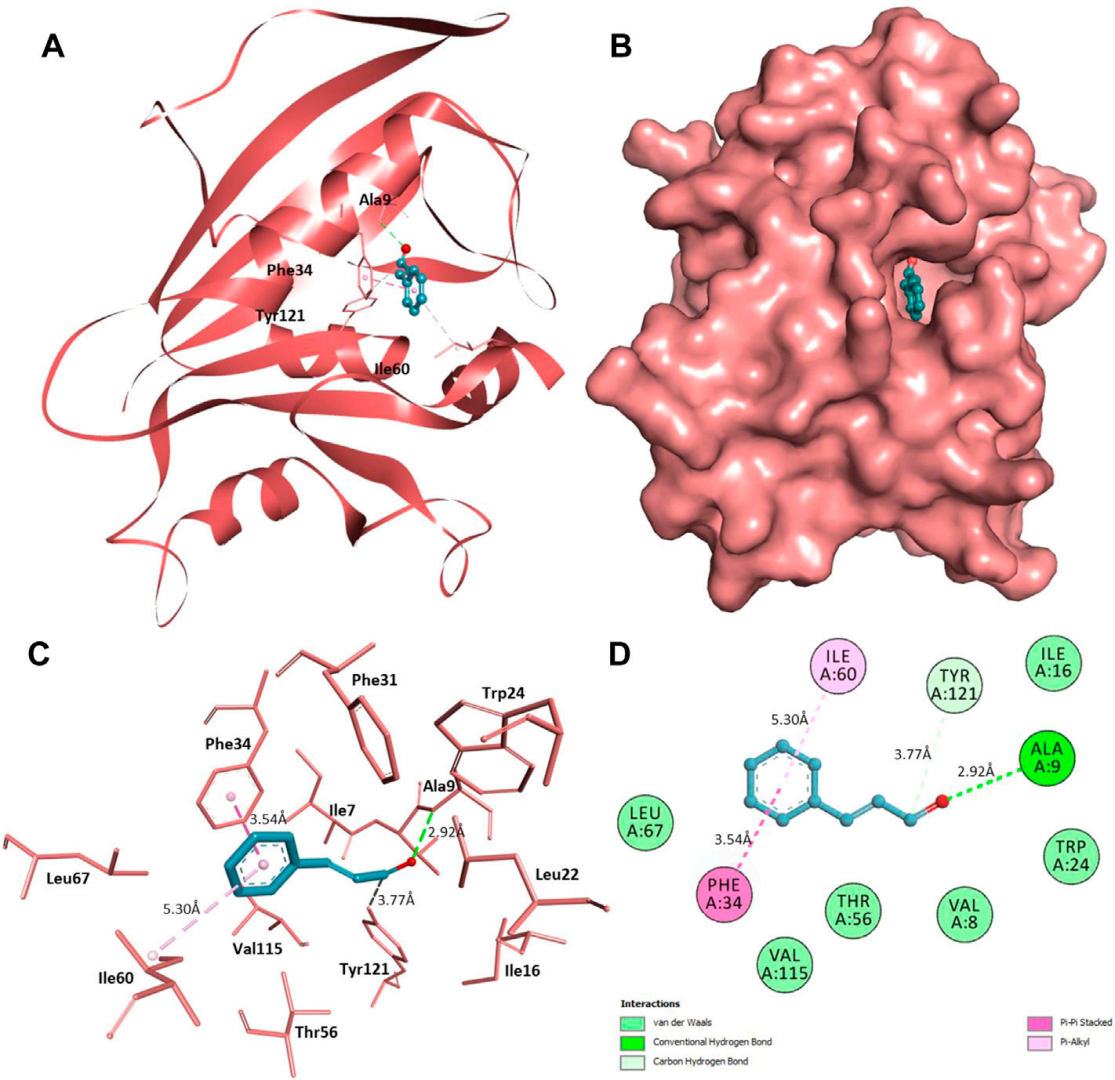
Interaction of CIN to DHFR **(A)** Cartoon representation of DHFR in-complexed with CIN **(B)** Surface view of DHFR in-complexed with CIN **(C)** 3D representation of interacting residues of DHFR with CIN **(D)** 2D plot of interacting residues of DHFR with CIN.

Furthermore, interaction analysis was carried out to analyze the binding prototype of CIN to MAPKP38α and DHFR (Table 2). In the case of MAPKP38α, it has been observed that the interaction between its catalytic residues, including Tyr35, Val38, Lys53, Met109, and Phe169, while in the case of DHFR, the interaction between Ala9, Phe34, Ile60, and Tyr121 with the small compound has been shown to stop or significant changes in their activity. CIN is present in the deep cavity of MAPKP38α and DHFR, showing several close interactions with their catalytic residues (Figure 9 and Figure 10).

**TABLE 2:** List of interacting residues of MAP kinase p38 alpha and DHFR to CIN.

S. No.	Target	Interacting Residues	
		Close contacts	Other soft interactions
1.	MAP kinase p38 alpha	Tyr35, Val38, Lys53, Met109, and Phe169	Val30, Val38, Ala51, Thr106, His107, Leu108, Gly170, and Leu171
2.	Dihydro Folate Reductase	Ala9, Phe34, Ile60, and Tyr121	Ile7, Ile16, Leu22, Trp24, Phe31, Thr56, Leu67, and Val115

Several residues of the substrate binding pocket are forming strong hydrogen bonds to the CIN and several van der Waals and other soft interactions to properly hold it in the binding cavity of MAPKP38α and DHFR (Figure 9 and Figure 10). Surface representations indicate that the CIN occupies the internal cavity of MAP Kinase p38 alpha and DHFR with appreciable affinity.

## Discussion

Chemotherapy, radiotherapy, and surgery are the currently available treatments for oral cancer. Chemotherapy drugs explore their ability to interfere with or inhibit the cell cycle by directly interacting with DNA or targeting proteins and enzymes required in cell cycle progression. Cisplatin and carboplatin are an example of drugs targeting DNA replication. 5-fluorouracil acts by inhibiting thymidylate synthase and incorporating its metabolites into RNA and DNA. Methotrexate is another example of a chemotherapeutic agent which inhibits DHFR and impairs the purines, thymidylate and certain amino acids. Radiation therapy for OSCC involves ionizing or high-energy photons to kill cancer cells. Surgical procedures are followed if the tumor is local and early (Gharat et al., 2016; Adeyemi and Choonara, 2022). The expression of EGFR and COX-2 is known to upregulate in oral cancer. So, EGFR and COX-2 inhibitors are used for treating OSCC (Li et al., 2020). In the late stages of OSCC, surgical procedures and radiotherapy fail to eliminate all the cancer cells. So, these therapies are used in combination with chemotherapy.

Further, chemotherapy comes with side effects as it can affect normal dividing cells also. There is no therapy available that gives a guaranteed treatment of OSCC. Reoccurrence after treatment is a major problem. So, discovering new therapies or upgrading traditional ones for oral cancer treatment is the need of the hour. Natural compounds are preferable candidates for medicine because of their availability, lower side effects and lower cost.

Based on consensus binding affinities, critical interactions and binding, CIN might act as a possible binding partner of MAPKP38α and DHFR, which can modulate their functional activity *via* decreasing the accessibility of substrate. CIN may have a high potential to inhibit their function, thereby acting as a putative therapeutic agent for several diseases.

The cell cytotoxicity and clonogenicity assays revealed the anti-tumor properties of CZE and CIN. Cell death possibly takes place through apoptosis, autophagy and necrosis. Flow cytometry analysis by AnnexinV staining and microscopic analysis by DAPI staining showed the induction of apoptosis in cancer cells after the treatment. Downregulating *NF-κB* and *Bcl-2* proteins could be the possible reason for the induction of apoptosis in cancer cells. In addition to *NF-κB* downregulation, CIN also induces the nuclear translocation of *NF-κB* i.e. from the nucleus to the cytoplasm, in oral cancer cells. The presence of autophagic vacuoles in cancer cells after treatment showed the induction of autophagy in cancer cells. Beclin-1 upregulation could play an important role in autophagy.

Flowcytometric analysis by PI staining clearly showed the cell cycle arrest in cancer cells after treatment. Further, we found cyclin D1 downregulation could be an important mechanism for CZE and CIN to arrest the cell cycle in the cancer cells. The property of CIN to arrest cell cycle arrest makes it a better candidate for chemotherapeutic drugs. COX-2 inhibitors act as non-steroidal anti-inflammatory drugs (NSAIDs). The COX-2 plays an important role in prostaglandins synthesis, and inflammation carcinogenesis. It is upregulated in oral cancer patients and peptide-mediated inhibition of COX-2 successfully inhibits growth in cancer cell lines (Kapoor et al., 2010). Our present study showed similar results of downregulation of COX-2 after treating CZE and CIN ultimately leading to tumor inhibition.

During the process of metastasis tumor, cells detach from the primary site, migrate to another site through the circulatory or lymphatic system, invade and get arrested in the secondary site. In this study, we used matrigel cell invasion assay to analyze the change in invasive properties of oral cancer cell lines after treatment of CZE and CIN. We found a significant reduction in invasion with both treatments. Further, the VEGF was downregulated after the treatment. VEGF has also been shown to promote angiogenesis (Zhang et al., 2000). Hence our study suggests the role of CZE and CIN in inhibiting malignancy and tumor progression.

PI3K/Akt/mTOR signaling pathway regulates cellular processes like proliferation, adhesion, migration, growth and apoptosis. This pathway is most frequently activated in cancer cancers that cross-talk with p53 and retinoblastoma pathways, leading to tumor progression (Yang et al., 2019). Activation of the PI3K/Akt/mTOR pathway can occur through mutations of PI3K, inactivation of the tumor suppressor phosphatase and tensin homolog (PTEN) and mutation of Akt (Hennessy et al., 2005). Point mutations in PIK3CA, the gene encoding the p110α subunit of PI3K, are among the most commonly demonstrated mutations in cancer (Samuels et al., 2004). PI3K/Akt/mTOR pathway could be exploited or targeted for efficiently treating various cancers. Previously our lab has documented the inhibition of cancer growth by using PI3K inhibitors (PI-828, PI-103, and PX-866) (Aggarwal et al., 2014; Aggarwal et al., 2015; Aggarwal and Das, 2016; Aggarwal et al., 2019). The present study demonstrated the downregulation of different components of PI3K/Akt/mTOR pathway in oral cancer cell lines treated with CZE and CIN. This implies that PI3K/Akt/mTOR inhibition could be one of the possible mechanisms by which CZE and CIN inhibit cancer growth and progression.

## Conclusion

We showed the inhibition of OSCC by CZE and CIN through various mechanisms, i.e., apoptosis, autophagy and cell cycle arrest. Further, for the first time, we have shown the binding of Cinnamaldehyde with MAP kinase p38 alpha and DHFR, which are key targets for inhibiting cancer. We explored PI3K/Akt/mTOR pathway for inhibiting oral cancer. Being a natural compound, easily available, lower cost, CIN could be a significant drug to treat OSCC. Its significance, while combined with other commercial drugs, needs further investigation.

## Data Availability

The original contributions presented in the study are included in the article/Supplementary Material, further inquiries can be directed to the corresponding authors.

## Abbreviations

CZE, Cinnamomum zeylanicum extract; CIN, Cinnamaldehyde; OSCC, Oral squamous cell carcinoma; DHFR, Dihydro Folate Reductase.

## References

[B1] Adeyemi S. A. Choonara Y. E. (2022). Current Advances in Cell Therapeutics: A Biomacromolecules Application Perspective. Expert Opin. Drug Deliv. 8, 2064844. 10.1080/17425247.2022.2064844 35395914

[B2] Aggarwal S. Das S. N. (2016). Garcinol Inhibits Tumour Cell Proliferation, Angiogenesis, Cell Cycle Progression and Induces Apoptosis via NF-Κb Inhibition in Oral Cancer. Tumour Biol. 37, 7175–7184. 10.1007/s13277-015-4583-8 26662963

[B3] Aggarwal S. Devaraja K. Sharma S. C. Das S. N. (2014). Expression of Vascular Endothelial Growth Factor (VEGF) in Patients with Oral Squamous Cell Carcinoma and its Clinical Significance. Clin. Chim. Acta 436, 35–40. 10.1016/j.cca.2014.04.027 24833243

[B4] Aggarwal S. John S. Sapra L. Sharma S. C. Das S. N. (2019). Targeted Disruption of PI3K/Akt/mTOR Signaling Pathway, via PI3K Inhibitors, Promotes Growth Inhibitory Effects in Oral Cancer Cells. Cancer Chemother. Pharmacol. 83, 451–461. 10.1007/s00280-018-3746-x 30519710

[B5] Aggarwal S. Sharma S. C. Das S. N. (2015). Galectin-1 and Galectin-3: Plausible Tumour Markers for Oral Squamous Cell Carcinoma and Suitable Targets for Screening High-Risk Population. Clin. Chim. Acta 442, 13–21. 10.1016/j.cca.2014.12.038 25578395

[B6] Albuquerque V. Q. Soares M. J. C. Matos M. N. C. Cavalcante R. M. B. Guerrero J. A. P. Soares Rodrigues T. H. (2021). Anti-Staphylococcal Activity of Cinnamomum Zeylanicum Essential Oil against Planktonic and Biofilm Cells Isolated from Canine Otological Infections. Antibiotics 11, 4. 10.3390/antibiotics11010004 35052881 PMC8773145

[B7] Alsayadi A. I. Abutaha N. Almutairi B. O. Al-Mekhlafi F. A. Wadaan M. A. (2022). Evaluating the Efficacy of an Innovative Herbal Formulation (HF6) on Different Human Cancer Cell Lines. Environ. Sci. Pollut. Res. Int. 6. 10.1007/s11356-022-19529-9 35249198

[B8] Anjum F. Mohammad T. Almalki A. A. Akhtar O. Abdullaev B. Hassan M. I. (2021). Phytoconstituents and Medicinal Plants for Anticancer Drug Discovery: Computational Identification of Potent Inhibitors of PIM1 Kinase. OMICS 25, 580–590. 10.1089/omi.2021.0107 34448628

[B9] Anwar S. Khan S. Shamsi A. Anjum F. Shafie A. Islam A. (2021). Structure-based Investigation of MARK4 Inhibitory Potential of Naringenin for Therapeutic Management of Cancer and Neurodegenerative Diseases. J. Cell. Biochem. 122, 1445–1459. 10.1002/jcb.30022 34121218

[B10] Anwar S. Shamsi A. Kar R. K. Queen A. Islam A. Ahmad F. (2020a). Structural and Biochemical Investigation of MARK4 Inhibitory Potential of Cholic Acid: Towards Therapeutic Implications in Neurodegenerative Diseases. Int. J. Biol. Macromol. 161, 596–604. 10.1016/j.ijbiomac.2020.06.078 32535203

[B11] Anwar S. Shamsi A. Shahbaaz M. Queen A. Khan P. Hasan G. M. (2020b). Rosmarinic Acid Exhibits Anticancer Effects via MARK4 Inhibition. Sci. Rep. 10, 10300. 10.1038/s41598-020-65648-z 32587267 PMC7316822

[B12] Anwar S. DasGupta D. Azum N. Alfaifi S. Y. M. Asiri A. M. Alhumaydhi F. A. (2022a). Inhibition of PDK3 by Artemisinin, a Repurposed Antimalarial Drug in Cancer Therapy. J. Mol. Liq. 355, 118928. 10.1016/j.molliq.2022.118928

[B13] Anwar S. DasGupta D. Shafie A. Alhumaydhi F. A. Alsagaby S. A. Shahwan M. (2022b). Implications of Tempol in Pyruvate Dehydrogenase Kinase 3 Targeted Anticancer Therapeutics: Computational, Spectroscopic, and Calorimetric Studies. J. Mol. Liq. 350, 118581. 10.1016/j.molliq.2022.118581

[B14] Anwar S. Khan S. Anjum F. Shamsi A. Khan P. Fatima H. (2022c). Myricetin Inhibits Breast and Lung Cancer Cells Proliferation via Inhibiting MARK4. J. Cell. Biochem. 123, 359–374. 10.1002/jcb.30176 34751461

[B15] Azevedo R. van Zeeland M. Raaijmakers H. Kazemier B. de Vlieg J. Oubrie A. (2012). X-ray Structure of P38α Bound to TAK-715: Comparison with Three Classic Inhibitors. Acta Crystallogr. D. Biol. Crystallogr. 68, 1041–1050. 10.1107/S090744491201997X 22868770

[B16] Braglia L. Zavatti M. Vinceti M. Martelli A. M. Marmiroli S. (2020). Deregulated PTEN/PI3K/AKT/mTOR Signaling in Prostate Cancer: Still a Potential Druggable Target? Biochim. Biophys. Acta Mol. Cell. Res. 1867, 118731. 10.1016/j.bbamcr.2020.118731 32360668

[B17] Bray F. Ren J. S. Masuyer E. Ferlay J. (2013). Global Estimates of Cancer Prevalence for 27 Sites in the Adult Population in 2008. Int. J. Cancer 132, 1133–1145. 10.1002/ijc.27711 22752881

[B18] Budiastuti D. Rosy Dwi N. Riesta P. Sukardiman R. (2021). Anti-Inflammatory Activity of Cinnamon Bark Oil (Cinnamomum Burmannii (Nees & T. Nees) Blume from Lombok Timur Indonesia. Pharmacogn. J. 13, 1005–1013. 10.5530/pj.2021.13.130

[B19] Chang S. Qin D. Wang L. Zhang M. Yan R. Zhao C. (2021). Preparation of Novel Cinnamaldehyde Derivative-BSA Nanoparticles with High Stability, Good Cell Penetrating Ability, and Promising Anticancer Activity. Colloids Surfaces A Physicochem. Eng. Aspects 624, 126765. 10.1016/j.colsurfa.2021.126765

[B20] Chowdhury C. R. Dey Chowdhury A. Shahnawaz K. Markus A. F. (2022). Level of Oral Cancer Awareness Among Indian Rural Population: A Possible Research Model Using Knowledge, Attitude and Practice (KAP) Intervention and its Utilisation in Low Resource Settings of LMICs. J. Oral Biol. Craniofac Res. 12, 154–160. 10.1016/j.jobcr.2021.10.008 34824968 PMC8604810

[B21] Chung C. H. Li J. Steuer C. E. Bhateja P. Johnson M. Masannat J. (2022). Phase II Multi-Institutional Clinical Trial Result of Concurrent Cetuximab and Nivolumab in Recurrent And/or Metastatic Head and Neck Squamous Cell Carcinoma. Clin. Cancer Res. 28. 10.1158/1078-0432.ccr-21-3849 PMC9167762 35344035

[B22] Dolado I. Swat A. Ajenjo N. De Vita G. Cuadrado A. Nebreda A. R. (2007). p38alpha MAP Kinase as a Sensor of Reactive Oxygen Species in Tumorigenesis. Cancer Cell. 11, 191–205. 10.1016/j.ccr.2006.12.013 17292829

[B23] Ferlay J. Colombet M. Soerjomataram I. Parkin D. M. Piñeros M. Znaor A. (2021). Cancer Statistics for the Year 2020: An Overview. Int. J. Cancer 149, 778–789. 10.1002/ijc.33588 33818764

[B24] García-Hernández L. García-Ortega M. B. Ruiz-Alcalá G. Carrillo E. Marchal J. A. García M. (2021). The P38 MAPK Components and Modulators as Biomarkers and Molecular Targets in Cancer. Int. J. Mol. Sci. 23, 370. 10.3390/ijms23010370 35008796 PMC8745478

[B25] Gharat S. A. Momin M. Bhavsar C. (2016). Oral Squamous Cell Carcinoma: Current Treatment Strategies and Nanotechnology-Based Approaches for Prevention and Therapy. Crit. Rev. Ther. Drug Carr. Syst. 33, 363–400. 10.1615/CritRevTherDrugCarrierSyst.2016016272 27910740

[B26] Gkouveris I. Nikitakis N. Sklavounou A. (2020). p38 Expression and Modulation of STAT3 Signaling in Oral Cancer. Pathol. Oncol. Res. 26, 183–192. 10.1007/s12253-018-0405-9 29564744

[B27] Hennessy B. T. Smith D. L. Ram P. T. Lu Y. Mills G. B. (2005). Exploiting the PI3K/AKT Pathway for Cancer Drug Discovery. Nat. Rev. Drug Discov. 4, 988–1004. 10.1038/nrd1902 16341064

[B28] Jairajpuri D. S. Hussain A. Nasreen K. Mohammad T. Anjum F. Tabish Rehman M. (2021). Identification of Natural Compounds as Potent Inhibitors of SARS-CoV-2 Main Protease Using Combined Docking and Molecular Dynamics Simulations. Saudi J. Biol. Sci. 28, 2423–2431. 10.1016/j.sjbs.2021.01.040 33526965 PMC7839507

[B29] Kapoor V. Singh A. K. Dey S. Sharma S. C. Das S. N. (2010). Circulating Cycloxygenase-2 in Patients with Tobacco-Related Intraoral Squamous Cell Carcinoma and Evaluation of its Peptide Inhibitors as Potential Antitumor Agent. J. Cancer Res. Clin. Oncol. 136, 1795–1804. 10.1007/s00432-010-0837-4 20213098 PMC11827827

[B30] Kennedy N. J. Cellurale C. Davis R. J. (2007). A Radical Role for P38 MAPK in Tumor Initiation. Cancer Cell. 11, 101–103. 10.1016/j.ccr.2007.01.009 17292820

[B31] Kumari A. Singh K. (2021). Evaluation of Prophylactic Efficacy of Cinnamaldehyde in Murine Model against Paradendryphiella Arenariae Mycotoxin Tenuazonic Acid-Induced Oxidative Stress and Organ Toxicity. Sci. Rep. 11, 021–98319. 10.1038/s41598-021-98319-8 PMC8484465 34593834

[B32] Kundu S. Dhar B. Das R. Laskar S. Singh S. A. Kapfo W. (2022). The Impact of Prehistoric Human Dispersals on the Presence of Tobacco-Related Oral Cancer in Northeast India. Gene 813, 146098. 10.1016/j.gene.2021.146098 34952175

[B33] Li S. Jiang M. Wang L. Yu S. (2020). Combined Chemotherapy with Cyclooxygenase-2 (COX-2) Inhibitors in Treating Human Cancers: Recent Advancement. Biomed. Pharmacother. 129, 110389. 10.1016/j.biopha.2020.110389 32540642

[B34] Li X. Liu H. Lv C. Du J. Lian F. Zhang S. (2022). Gypenoside-Induced Apoptosis via the PI3K/AKT/mTOR Signaling Pathway in Bladder Cancer. Biomed. Res. Int. 29, 9304552. 10.1155/2022/9304552 PMC8984741 35402614

[B35] Marques A. E. M. Borges G. A. Viesi do Nascimento Filho C. H. Vianna L. M. S. Ramos D. D. A. R. Castilho R. M. (2022). Expression Profile of the PI3K-AKT-mTOR Pathway in Head and Neck Squamous Cell Carcinoma: Data from Brazilian Population. Oral Surg. Oral Med. Oral Pathol. Oral Radiol. 133, 453–461. 10.1016/j.oooo.2021.10.020 35153184

[B36] Mohammad T. Mathur Y. Hassan M. I. (2021). InstaDock: A Single-Click Graphical User Interface for Molecular Docking-Based Virtual High-Throughput Screening. Brief. Bioinform 22, bbaa279. 10.1093/bib/bbaa279 33105480

[B37] Mohammad T. Siddiqui S. Shamsi A. Alajmi M. F. Hussain A. Islam A. (2020). Virtual Screening Approach to Identify High-Affinity Inhibitors of Serum and Glucocorticoid-Regulated Kinase 1 Among Bioactive Natural Products: Combined Molecular Docking and Simulation Studies. Molecules 25, 823. 10.3390/molecules25040823 PMC7070812 32070031

[B38] Moreno E. K. G. de Macêdo I. Y. L. Batista E. A. Machado F. B. Santos G. R. Andrade D. M. L. (2022). Evaluation of Antioxidant Potential of Commercial Cinnamon Samples and its Vasculature Effects. Oxid. Med. Cell. Longev. 23, 1992039. 10.1155/2022/1992039 PMC8967587 35368871

[B39] Nandi S. Mandal A. Chhebbi M. (2021). The Prevalence and Clinicopathological Correlation of Human Papillomavirus in Head and Neck Squamous Cell Carcinoma in India: A Systematic Review Article. Cancer Treat. Res. Commun. 26, 100301. 10.1016/j.ctarc.2020.100301 33401132

[B40] Naqvi A. A. T. Hassan M. I. (2016). Methods for Docking and Drug Designing. Oncol. Break. Res. Pract. 2-2, 876–890. 10.4018/978-1-5225-0115-2.ch002

[B41] Naqvi A. A. T. Jairajpuri D. S. Noman O. M. A. Hussain A. Islam A. Ahmad F. (2020). Evaluation of Pyrazolopyrimidine Derivatives as Microtubule Affinity Regulating Kinase 4 Inhibitors: Towards Therapeutic Management of Alzheimer's Disease. J. Biomol. Struct. Dyn. 38, 3892–3907. 10.1080/07391102.2019.1666745 31512980

[B42] Naqvi A. A. T. Mohammad T. Hasan G. M. Hassan M. I. (2018). Advancements in Docking and Molecular Dynamics Simulations towards Ligand-Receptor Interactions and Structure-Function Relationships. Curr. Top. Med. Chem. 18, 1755–1768. 10.2174/1568026618666181025114157 30360721

[B43] Neto J. G. O. Boechat S. K. Romão J. S. Kuhnert L. R. B. Pazos-Moura C. C. Oliveira K. J. (2022). Cinnamaldehyde Treatment during Adolescence Improves White and Brown Adipose Tissue Metabolism in a Male Rat Model of Early Obesity. Food Funct. 13, 3405–3418. 10.1039/d1fo03871k 35230374

[B44] Qaid M. M. Al-Mufarrej S. I. Azzam M. M. Al-Garadi M. A. (2021). Anticoccidial Effectivity of a Traditional Medicinal Plant, Cinnamomum Verum, in Broiler Chickens Infected with Eimeria Tenella. Poult. Sci. 100, 9. 10.1016/j.psj.2020.11.071 33518353 PMC7936149

[B45] Queen A. Khan P. Idrees D. Azam A. Hassan M. I. (2018). Biological Evaluation of P-Toluene Sulphonylhydrazone as Carbonic Anhydrase IX Inhibitors: An Approach to Fight Hypoxia-Induced Tumors. Int. J. Biol. Macromol. 106, 840–850. 10.1016/j.ijbiomac.2017.08.082 28830777

[B46] Roy S. Khan S. Jairajpuri D. S. Hussain A. Alajmi M. F. Islam A. (2021). Investigation of Sphingosine Kinase 1 Inhibitory Potential of Cinchonine and Colcemid Targeting Anticancer Therapy. J. Biomol. Struct. Dyn. 10, 1–13. 10.1080/07391102.2021.1882341 33565370

[B47] Samuels Y. Wang Z. Bardelli A. Silliman N. Ptak J. Szabo S. (2004). High Frequency of Mutations of the PIK3CA Gene in Human Cancers. Science 304, 554. 10.1126/science.1096502 15016963

[B48] Shamshad H. Bakri R. Mirza A. Z. (2022). Dihydrofolate Reductase, Thymidylate Synthase, and Serine Hydroxy Methyltransferase: Successful Targets against Some Infectious Diseases. Mol. Biol. Rep. 7, 1–33. 10.1007/s11033-022-07266-8 PMC8898753 35253073

[B49] Song X. Wei C. Li X. (2022). The Signaling Pathways Associated with Breast Cancer Bone Metastasis. Front. Oncol. 12, 855609. 10.3389/fonc.2022.855609 35372035 PMC8965611

[B50] Srinivasan B. Tonddast-Navaei S. Roy A. Zhou H. Skolnick J. (2019). Chemical Space of *Escherichia coli* Dihydrofolate Reductase Inhibitors: New Approaches for Discovering Novel Drugs for Old Bugs. Med. Res. Rev. 39, 684–705. 10.1002/med.21538 30192413 PMC6370515

[B51] Stevens N. Allred K. (2022). Antidiabetic Potential of Volatile Cinnamon Oil: A Review and Exploration of Mechanisms Using In Silico Molecular Docking Simulations. Molecules 27, 853. 10.3390/molecules27030853 35164117 PMC8840343

[B52] Yang J. Nie J. Ma X. Wei Y. Peng Y. Wei X. (2019). Targeting PI3K in Cancer: Mechanisms and Advances in Clinical Trials. Mol. Cancer 18, 26. 10.1186/s12943-019-0954-x 30782187 PMC6379961

[B53] Yu C. H. Chu S. C. Yang S. F. Hsieh Y. S. Lee C. Y. Chen P. N. (2019). Induction of Apoptotic but Not Autophagic Cell Death by Cinnamomum cassia Extracts on Human Oral Cancer Cells. J. Cell. Physiol. 234, 5289–5303. 10.1002/jcp.27338 30317581

[B54] Zaware N. Kisliuk R. Bastian A. Ihnat M. A. Gangjee A. (2017). Synthesis and Evaluation of 5-(arylthio)-9h-Pyrimido[4,5-B]indole-2,4-Diamines as Receptor Tyrosine Kinase and Thymidylate Synthase Inhibitors and as Antitumor Agents. Bioorg Med. Chem. Lett. 27, 1602–1607. 10.1016/j.bmcl.2017.02.018 28258797 PMC5398096

[B55] Zeyen L. Seternes O. M. Mikkola I. (2022). Crosstalk between P38 MAPK and GR Signaling. Int. J. Mol. Sci. 23, 3322. 10.3390/ijms23063322 35328742 PMC8953609

[B56] Zhang Z. G. Zhang L. Jiang Q. Zhang R. Davies K. Powers C. (2000). VEGF Enhances Angiogenesis and Promotes Blood-Brain Barrier Leakage in the Ischemic Brain. J. Clin. Invest. 106, 829–838. 10.1172/JCI9369 11018070 PMC517814

