# Supplementary material for: Cinnamomum zeylanicum Extract and its Bioactive Component Cinnamaldehyde Show Anti-Tumor Effects via Inhibition of Multiple Cellular Pathways
Source: Front Pharmacol. 2022 Jun 2;13:918479. doi: 10.3389/fphar.2022.918479 (PMC9237655; doi:10.3389/fphar.2022.918479)
Supplement: Supplementary file 1 [file Presentation1.ppt]

## Slide 1
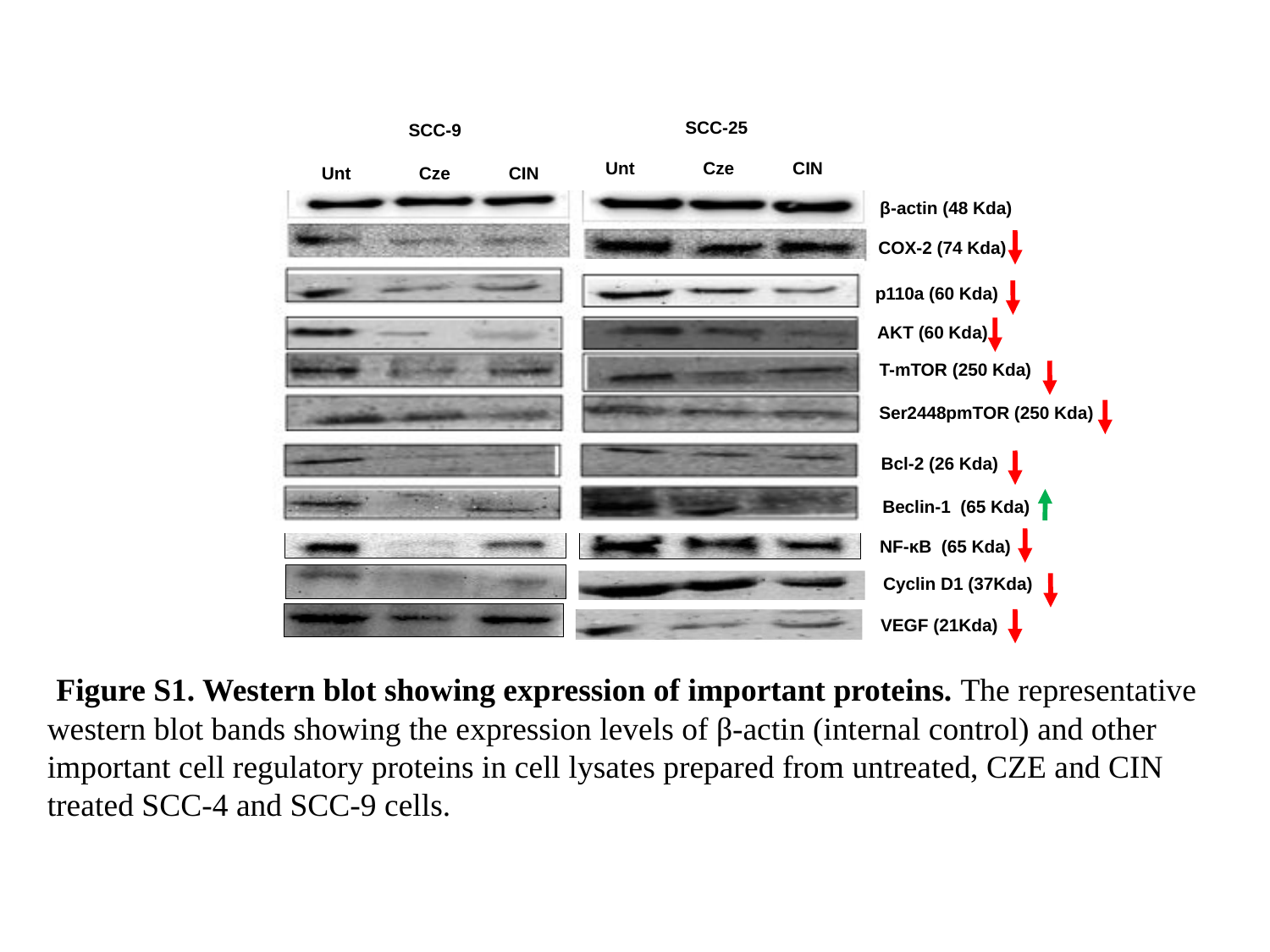

SCC-25
SCC-9
 Unt Cze CIN
 Unt Cze CIN
β-actin (48 Kda)
COX-2 (74 Kda)
p110a (60 Kda)
AKT (60 Kda)
T-mTOR (250 Kda)
Ser2448pmTOR (250 Kda)
Bcl-2 (26 Kda)
Beclin-1 (65 Kda)
NF-ĸB (65 Kda)
Cyclin D1 (37Kda)
VEGF (21Kda)
 Figure S1. Western blot showing expression of important proteins. The representative western blot bands showing the expression levels of β-actin (internal control) and other important cell regulatory proteins in cell lysates prepared from untreated, CZE and CIN treated SCC-4 and SCC-9 cells.
